# ERCC1 19007 Polymorphism in Greek Patients with Advanced Urothelial Cancer Treated with Platinum-Based Chemotherapy: Effect of the Changing Treatment Paradigm: A Cohort Study by the Hellenic GU Cancer Group

**DOI:** 10.3390/curroncol28060380

**Published:** 2021-11-05

**Authors:** Aristotelis Bamias, Konstantinos Koutsoukos, Nikos Gavalas, Roubini Zakopoulou, Kimon Tzannis, Nikos Dedes, Anna Boulouta, Charalampos Fragkoulis, Eythymios Kostouros, Athanasios Dellis, Iraklis Mitsogiannis, Ioannis Adamakis, Ioannis Anastasiou, Andreas Skolarikos, Athanasios Papatsoris, Konstantinos Stravodimos, Nikolaos Ferakis, Stamatina Pagoni, Konstantinos Ntoumas, Dionysios Mitropoulos, Charalambos Deliveliotis, Constantinos A. Constantinides, Meletios A. Dimopoulos

**Affiliations:** 1 2nd Propaedeutic Department of Internal Medicine, National & Kapodistrian University of Athens, ATTIKON University Hospital, 12462 Athens, Greece; Rzakopoul@gmail.com (R.Z.); kimon.tzannis@gmail.com (K.T.); annitaboulouta@gmail.com (A.B.); 2 Hellenic Genito-Urinary Cancer Group, 89 Evrou St, 11527 Athens, Greece; koutsoukos.k@gmail.com (K.K.); ngavalas@med.uoa.gr (N.G.); harisfrag@yahoo.gr (C.F.); ntoumask@yahoo.com (K.N.); mdimop@med.uoa.gr (M.A.D.); 3 Department of Clinical Therapeutics, National & Kapodistrian University of Athens, ALEXANDRA Hospital, 11528 Athens, Greece; dedes.nik.95@gmail.com; 4 Department of Urology, General Hospital of Athens “G. Gennimatas”, 11527 Athens, Greece; 5 3rd Department of Internal Medicine, General Hospital of Athens “G. Gennimatas”, 11527 Athens, Greece; ekostouros@gmail.com (E.K.); pagonimatina@gmail.com (S.P.); 6 2nd Department of Surgery, Aretaieion Academic Hospital, School of Medicine, National & Kapodistrian University of Athens, 15772 Athens, Greece; aedellis@gmail.com; 7 2nd Department of Urology, Sismanoglio Hospital, National & Kapodistrian University of Athens, 15772 Athens, Greece; imitsog@med.uoa.gr (I.M.); andskol@yahoo.com (A.S.); agpapatsoris@yahoo.gr (A.P.); chdeliveli@gmail.com (C.D.); 8 1st University Urology Clinic, Laiko Hospital, University of Athens, 11527 Athens, Greece; iadamakis@med.uoa.gr (I.A.); ianastas@med.uoa.gr (I.A.); kstravd@med.uoa.gr (K.S.); dmp@otenet.gr (D.M.); ckonstan@med.uoa.gr (C.A.C.); 9 Department of Urology, Korgialenio Benakio Hellenic Red Cross Hospital, 11526 Athens, Greece; ferakis@otenet.gr

**Keywords:** ERCC1, bladder cancer, chemotherapy, immunotherapy, vinflunine

## Abstract

We previously showed that ERCC1 19007 C>T polymorphism was associated with cancer-specific survival (CSS) after platinum-based chemotherapy in patients with advanced urothelial cancer (aUC). We aimed to confirm this association in a different cohort of patients. Genotyping of the 19007C>T polymorphism was carried out by polymerase chain reaction (PCR) amplification and restriction fragment length polymorphism (RFLP) in 98 aUC patients, treated with platinum-based chemotherapy. Median age of the patients was 68.8, 13.3% of them were female, 90.8% had ECOG PS of 0 or 1, and 48% received cisplatin-based chemotherapy. In addition to chemotherapy, 32.7% of the patients received immunotherapy, and 19.4% vinflunine. Eighty-one patients (82.7%) were carriers of the 19007T polymorphic allele: 46 (46.9%) were heterozygotes, and 35 (35.7%) were homozygotes. The ERCC1 polymorphism was not associated with CSS, progression-free (PFS), or overall (OS) survival in the total population. Nevertheless, there was a significant interaction between the prognostic significance of ERCC1 polymorphism and the use of modern immunotherapy: the T allele was associated with worse outcome in patients who received chemotherapy only, while this association was lost in patients who received both chemotherapy and immune checkpoint inhibitors. Our study suggests that novel therapies may influence the significance of ERCC1 polymorphism in patients with aUC. Its determination may be useful in the changing treatment landscape of the disease.

## 1. Introduction

Urothelial cancer (UC) is a common malignancy in Greece. With an age-standardized rate of 21.2 cases/100,000, Greece rates second in the world for both sexes and first for men, with a rate of 40.4 [1]. Most cases present as superficial cancers, requiring only local treatment. Nevertheless, 25% of UCs are muscle invasive (MIUC), while 5% present with locally advanced (inoperable) or metastatic disease, usually referred to as advanced urothelial cancer (aUC). Muscle invasion is a decisive prognostic factor associated with increased risk of death from UC. In addition, approximately half of the patients undergoing surgery for invasive disease will relapse. Such a development is also associated with unfavorable prognosis.

Combination, platinum-based chemotherapy has long been the mainstay of systemic therapy for MIUC, aUC, and relapsed UC. Urothelial cancer is a chemosensitive neoplasm. Long experience, initially with the combination of methotrexate, vinblastine, doxorubicin, and cisplatin (MVAC), showed response rates (RRs) of more than 50%, three-year survival of 20–25%, and median survival in excess of 1 year [2,3,4]. This efficacy was reproduced by other regimes, some of which proved more patient-friendly than classic MVAC [3,5,6]. Importantly, long-term progression-free survival was shown to be possible among patients treated with cisplatin-based chemotherapy, had Karnofsky performance status (PS) ≥ 80%, and only local and/or lymph node disease (LND) [7,8]. Nevertheless, still most patients experience progression of the disease. Although recent advances in the treatment of progressing UC with immunotherapy, antibody–drug conjugates, and inhibitors of the fibroblast growth factor receptor 3 (FGFR3) showed that a minority of patients can achieve long-term remission [9,10,11,12,13], relapse after systemic chemotherapy is usually associated with swift deterioration. Consequently, it is critical to identify factors linked with chemotherapy resistance and methods for circumventing this limitation. The nucleotide excision repair (NER) complex, a highly conserved multiprotein DNA repair structure, is one important mechanism resulting in resistance to platinum compounds. The NER complex acts as an enhanced cellular defense mechanism against platinum-induced DNA adduct formation. ERCC1, the major component of NER, forms a highly active catalytic heterodimer structure with the XPF enzyme that dissociates platinum-induced DNA adducts from genomic DNA [14,15,16]. Increased ERCC1 intracellular accumulation has been associated with increased clinical resistance to platinum-based chemotherapy [17,18,19]. Specifically in bladder cancer, reduced ERCC1 mRNA expression was correlated with increased cisplatin efficacy [20,21,22].

We previously investigated the relationship between two frequent ERCC1 single-nucleotide polymorphisms (SNPs), 19007C>T (rs11615) and 8092C>A (rs3212986), with outcomes in patients with aUC treated with platinum-based chemotherapy [23]. We found that the 19007C>T polymorphism could be a useful prognostic marker in this setting. Two frequent ERCC1 SNPs have been hypothesized to represent tumor activity. 19007C>T (rs11615) is situated in exon 4 of the gene and is linked with a change in the constitutional codon (AAC) of asparagine (N) to another codon (AAT), which has a functional effect on translational capacity (also referred to as N118N polymorphism). This polymorphism is associated with decreased levels of ERCC1 produced by basic and platinum agents, decreased NER complex activity, and decreased resistance to platinum action [24]. It has been associated with greater baseline and platinum-induced ERCC1 protein levels, enhanced NER complex activity, and increased resistance to platinum-based regimens. In several tumor types, such as non-small-cell lung, colorectal, ovarian, and head and neck cancer, associations between the aforementioned ERCC1 SNPs and clinical response have been reported [25,26,27,28,29]. 

We previously showed that the 19007C>T polymorphism, especially in its homozygotic state, but not the 8092C>A one, could be a useful prognostic marker in advanced UC treated with platinum-based chemotherapy [23]. The use of this factor refined the traditional prognostic algorithm, based on PS and site of metastases. We, therefore, aimed to confirm this finding in a different cohort of patients. This is particularly relevant in the context of the new treatment paradigm for aUC, with novel therapies prolonging survival in the post-platinum setting now being used in routine practice. Our analysis did not confirm the prognostic significance of the 19007C/T polymorphism. Subgroup analyses suggested that the utilization of novel therapies may, in fact, be responsible for not reproducing our previous findings.

## 2. Materials and Methods

### 2.1. Patients

Following our previous publication [23], we started collecting blood samples for DNA extraction prospectively from all patients with histologically confirmed UC, scheduled to start first-line chemotherapy in our institution. All patients gave their institutional review board (IRB)-approved written consent for the use of biological material and information from their medical examination. Blood was stored at −80 °C until processing. Patients selected for this study had advanced UC (clinical stage IIIB–IV) and were treated with platinum-based chemotherapy, either as first-line for advanced/metastatic disease or as neoadjuvant or adjuvant chemotherapy if recurrence had occurred within 12 months after neoadjuvant/adjuvant chemotherapy. Patients receiving treatment for advanced disease are usually followed up every 3 months with CT of abdomen and pelvis. Additional imaging is used according to the site of disease. The analysis was also IRB-approved. The genetic analysis was carried out in a blinded way at the laboratory of the Department of Clinical Therapeutics at Alexandra University Hospital (Athens, Greece). Information regarding their medical history and laboratory findings was entered in an anonymous fashion in the advanced UC database of the Hellenic Genito-Urinary Cancer Group. 

### 2.2. PCR Amplification

Genomic DNA was extracted using 1 ml of whole blood per patient under strictly sterile conditions, using the Invisorb Spin Blood Midi Kit (Stratec Molecular GmbH, Berlin, Germany) according to the manufacturer’s instructions. Purified DNA concentration ranged between 10 and 95 ng/μL. We used 5–10 μL per sample in a nested or 126 semi-nested PCR amplification, thus producing a high yield of the gene region containing the polymorphism under investigation. Briefly, amplification by nested PCR was performed using the following primers: outer forward primer 5′ –TGCAAGAAGAGGTGGAGGAGG-3′ (melting temperature [Tm] 60 °C), outer reverse primer 5′ -CTCCAGCTCTTGTTGCTCTG-3′ (Tm 56 ᵒC), forward nested primer 5′ -CTGTGGTTATCAAGGGTCATC-3′ (Tm 56 ᵒC), and reverse nested primer 5′ -TGGGCACCTCCAGGCCAAGA-3′ (Tm 60 ᵒC). The PCR conditions for each round, respectively, were: in the first round, one cycle for 1 min at 94 ᵒC, 35 cycles (for 1 min at 94 ᵒC, 1 min at 56 ᵒC, and 1 min at 72 ᵒC), followed by one cycle for 7 min at 72 ᵒC. The same conditions were applied for the nested round, with the exception of the total number of cycles allowed, which was 30 rather than 35. In both rounds, each DNA reaction mixture contained 0.5 μg of DNA/25 μL of final reaction volume. The PCR system used was Colorless Go Taq Flexi DNA Polymerase (Promega, Madison, WI, USA).

### 2.3. RFLP

Nested PCR products containing the 19007C>T SNP were digested overnight using BsrDI (New England Biolabs, Ipswich, MA, USA) as per the manufacturer’s instructions, leading to C/C (333 bp), C/T (333 bp, 242 p and 91 bp), and T/T (242 bp, 91 bp). 

### 2.4. Statistics

This was a retrospective study. Cancer-specific survival (CSS) was calculated as the time from initiation of first-line treatment to death from UC or the date of the last contact for alive patients. Patients that died of unrelated causes to the disease were censored at the time of their death. Overall survival (OS) was calculated as the time from initiation of treatment to death or the date of the last contact for alive patients. Progression-free survival (PFS) was calculated as the time from first-line treatment commencement to the date of recurrence, death, or last contact for non-relapsed patients. The association of the studied polymorphisms with clinical characteristics was evaluated using parametric or non-parametric statistical tests (chi-squared test, Fisher’s exact test, ANOVA, Kruskal–Wallis test). Plots of the Kaplan–Meier estimators for the studied polymorphism categories and other baseline characteristics are presented. The log-rank test was used to compare the survival distributions of subgroups; the stratified log-rank test was used to account for the differences in genotypes between subgroups. The factors studied for prognostic significance were: age (≤68 vs. >68), gender, body mass index (BMI), body surface area (BSA), treatment (cisplatin vs. carboplatin-based chemotherapy, immunotherapy yes vs. no, vinflunine yes vs. no), lines of treatment for aUC (≥2 lines vs. 1), Eastern Cooperative Oncology Group (ECOG) PS (0 vs. 1, 2), disease status (locally advanced [primary site and/or LND at any site] vs. distant metastases [any other site]), and Memorial Sloan-Kettering Cancer Center (MSKCC) risk stratification [7]. Specific populations studied included patients treated with immunotherapy and patients treated with vinflunine. Interaction terms between genotypes and other prognostic factors were included in Cox regression models. A *p*-value less than 0.05 was considered statistically significant. All analyses were carried out in statistical software STATA 17.0 SE (StataCorp LLC, College Station, TX, USA). 

## 3. Results

### 3.1. Patients

We identified 115 patients with histologically confirmed UC from whom pre-treatment blood samples were available. Seventeen patients were excluded from further analyses due to lack of clinical information (*n* = 1), no evidence of advanced or metastatic disease (*n* = 11), and no treatment with platinum-based chemotherapy (*n* = 5). Therefore, 98 patients with locally advanced or metastatic disease, treated from October 2009 to May 2017, were included in our analysis (Figure S1). Table 1 depicts their demographic and clinical characteristics, as well as the frequency distribution of the studied genotype and first-line therapy for advanced/metastatic disease. Bladder was the primary site in 83.7% of the cases. The majority of patients had ECOG PS 0 or 1 (90.8%), while 46.9% of them had distant metastases, and 49% belonged to the low-risk category according to MSKCC classification. All patients received platinum-based chemotherapy: 57 received cisplatin, and 41 carboplatin. In most cases, platinum-based chemotherapy was used as first-line therapy for advanced/metastatic disease. Ten patients received platinum-based chemotherapy either as neoadjuvant or as adjuvant treatment and received other therapy as first-line treatment. A detailed list of first-line therapy can be found in the Supplementary material (Table S1). Thirty-two patients (32.7%) received ICIs, while 19 patients (19.4%) received vinflunine at some point in the course of advanced/metastatic disease. 

### 3.2. ERCC1 19007 C>T Polymorphism

The frequency of ERCC1 19007 C>T polymorphisms was: T/T, 35 (35.7%), C/T, 46 (46.9%), C/C, 17 (17.4%). The T/T genotype was associated with more frequent distant metastases (62.9% vs. 34.8% and 47.1% for C/T and C/C, respectively), while fewer patients were categorized as low risk according to MSKCC criteria (31.4% vs. 60.9% and 52.9% for C/T and C/C, respectively) (Table 1). On the contrary, no association of any genotype with ECOG PS was observed.

### 3.3. Correlation of SNPs with CSS, OS, PFS, and Tumor Response

#### 3.3.1. Whole Population

**Figure 1:**
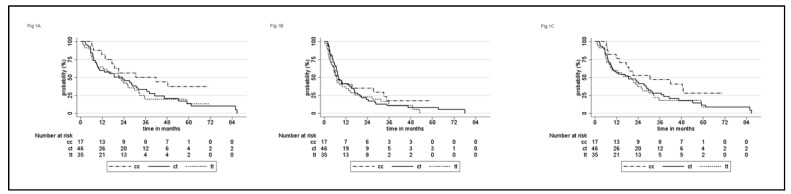
Cancer-specific (**A**), progression-free (**B**), and overall (**C**) survival of 98 patients with advanced urothelial cancer according to ERCC1 C19007T polymorphism.

Survival details for the entire population and according to each genotype are shown in Table 2. During a median follow-up of 62.9 months (95% CI 49.4–71.4), 90 patients (91.8%) relapsed, and 84 (85.7%) died, of which 7 patients died of causes unrelated to the disease. Median CSS was 22.7 months (95% CI: 15.8–30), median PFS was 7.3 months (95% CI: 6.2–10), and median OS 19.8 months (95% CI: 12.6–26.3). There was no correlation between ERCC1 19007 polymorphism with CSS, PFS, or OS (Figure 1). This lack of correlation was observed also after stratification for MSKCC risk category. A better Eastern Cooperative Oncology Group PS and la ower MSKCC risk group were associated with improved CSS (Table 2). Similar correlations were observed for PFS and OS (Tables S2 and S3)

#### 3.3.2. Subgroup Analyses

Since the type of therapy may impact on outcomes, we studied the interaction of ERCC1 SNPs with the platinum compound (cisplatin vs. carboplatin), immunotherapy (yes vs. no), and vinflunine treatment (yes vs. no) regarding CSS, OS, and PFS. 

**Figure 2:**
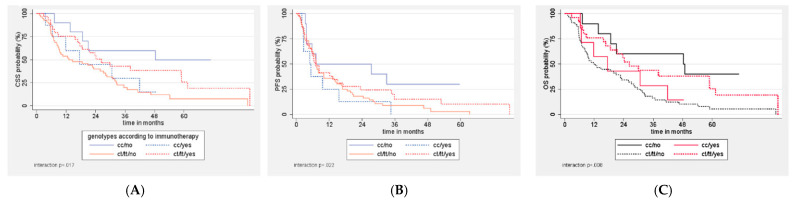
Correlation of ERCC1 genotypes with cancer-specific (**A**), progression-free (**B**), and overall (**C**) survival of the 98 patients with advanced urothelial cancer according to the administration of immunotherapy.

There was no interaction of ERCC1 SNPs with platinum compound or vinflunine treatment. On the contrary, there was a significant interaction between SNPs and the use of immunotherapy in CSS (*p* = 0.029), PFS (*p* = 0.018), and OS (*p* = 0.014). When patients who received immunotherapy were excluded (*n* = 32), the C/C genotype was associated with significantly longer CSS and OS compared to the other two genotypes (Table 3). These associations were retained after stratification for MSKCC risk category. On the contrary, among the 32 patients who received immunotherapy, an inverse numerical trend was observed, but without statistical significance (Table 3). Figure 2 shows the inverse correlation of CC vs. CT/TT genotype with CSS, PFS, and OS according to the administration of immunotherapy. 

## 4. Discussion

We report the second study on ERCC1 polymorphisms in Greek patients with advanced urothelial cancer. The frequency of the 19007T polymorphic allele was slightly higher than that reported in our first study (82% vs. 69%) [23]. Median CSS, PFS, OS, and RR were within the expected values for a population with the characteristics of that included in our study. Nevertheless, the associations of the different genotypes with outcome in the whole population were not confirmed. Our results suggest that this might be due to the changing landscape of treatment of aUC.

When analyses were restricted to a chemotherapy-only treated population, there was a significant association of ERCC1 SNPs with CSS, PFS, and OS: the C/C genotype was associated with significant improvement compared to the C/T or T/T genotype. This is in concert with previous reports on human malignancies, including urothelial cancer, treated with platinum compounds [24,30]. Genetic polymorphism may affect structure, function, stability, and folding of proteins. Consequently, polymorphism in the ERCC1 genes could affect its expression, which has been shown to be correlated with outcomes of patients with aUC treated with chemotherapy [25].

The association described above was lost when patients who had received ICIs were also included. Further investigation of this finding revealed an interaction between immunotherapy and genotype association with CSS, PFS, and OS: only immunotherapy benefited patients with the C/T or T/T genotype. This interaction was independent of the most established prognostic algorithm in aUC, namely, the MSKCC stratification. Although our subgroup analyses were limited by the small number of patients included, we believe that our results support that the role of ERCC1 in the evolving treatment paradigm of auC deserves further investigation. Anti-PD1 and anti-PDL1 agents have revolutionized the treatment of aUC [26]. Nevertheless, only about 20% of the treated patients achieve long-term remission of their disease. Intense research on biomarker-driven selection for immunotherapy of aUC is ongoing [27]. Taking into consideration that the optimal use of both platinum-based chemotherapy and ICI in the treatment paradigm of this disease is still under investigation, our results may be viewed as generating important research hypotheses regarding the use of ERCC1 in our armamentarium for proper patient selection for chemotherapy, immunotherapy, or both. Research on the role of ERCC1 activity in the efficacy of modern immunotherapy is limited, while it is completely lacking in aUC. In a recent report, the T/T genotype was associated with improved OS and PFS in patients with non-small-cell lung cancer [28], which is partially in concert with our findings.

The mechanism underlying the suggested differential effect of ERCC1 T19007C polymorphism on chemotherapy and immunotherapy is unclear. Again, the association of polymorphism with ERCC1 tumoral levels may be of importance. Low levels of ERCC1 have been shown to be correlated with high tumor mutational burden [29], which confers favorable response to immunotherapy in aUC treatment [31]. This potential dual predictive value of ERCC1, towards both chemotherapy and immunotherapy, makes it particularly attractive. It could be, for example, postulated that a tumor with low levels of ERCC1 would be ideally treated with a combination (simultaneous or sequential) of platinum-based chemotherapy and ICI. The recent OS benefit achieved by avelumab maintenance in patients not progressing after chemotherapy [32] supports this hypothesis. It is plausible that responders to chemotherapy may represent a population enriched for low ERCC1, which also predicts favorable response to avelumab.

## 5. Conclusions

Markers of chemotherapy resistance, such as ERCC1 SNPs, may be valuable in the current treatment paradigm of aUC.

## Figures and Tables

**Table 1:** Baseline characteristics of the 98 patients analyzed: association with the studied genotypes.

Characteristic	Total	ERCC1 19007 Genotypes			
		C/C	C/T	T/T	*p*
		Median (25th–75th percentile)			
Age	68.8 (63–73.7)	68.7 (59.1–75.4)	69.1 (64.6–73.7)	68.7 (58.8–73.4)	0.94 └
BMI	25.9 (23.1–29)	24.3 (23.4–29.4)	25.9 (23.5–29.1)	27 (22.8–28)	0.85 └
BSA	1.9 (1.8–2)	1.9 (1.8–2)	1.9 (1.8–2)	1.9 (1.8–2)	0.78 ┘
			*n* (%)		
Gender					0.33 ⅟
Female	13 (13.3)	2 (11.8)	4 (8.7)	7 (20)	
Male	85 (86.7)	15 (88.2)	42 (91.3)	28 (80)	
Primary site					0.26 ⅟
Bladder	82 (83.7)	12 (70.6)	40 (87)	30 (85.7)	
Pelvis	10 (10.1)	3 (17.6)	4 (8.7)	3 (8.6)	
Ureter	3 (3.1)	0 (0)	1 (2.2)	2 (5.7)	
Urethra	3 (3.1)	2 (11.8)	1 (2.2)	0 (0)	
ECOG PS					0.103 ⅟
0	56 (57.1)	9 (52.9)	24 (52.2)	23 (65.7)	
1	33 (33.7)	8 (47.1)	19 (41.3)	6 (17.1)	
2	6 (6.2)	0 (0)	3 (6.5)	3 (8.6)	
3	2 (2)	0 (0)	0 (0)	2 (5.7)	
missing	1 (1)	0 (0)	0 (0)	1 (2.9)	
Disease status					0.043
Distant metastases	46 (46.9)	8 (47.1)	16 (34.8)	22 (62.9)	
Locally advanced	52 (53.1)	9 (52.9)	30 (65.2)	13 (37.1)	
MSKCC risk *					0.079 ⅟
Low	48 (49)	9 (52.9)	28 (60.9)	11 (31.4)	
Intermediate	45 (45.9)	8 (47.1)	17 (37)	20 (57.1)	
High	4 (4.1)	0 (0)	1 (2.2)	3 (8.6)	
missing	1 (1)	0 (0)	0 (0)	1 (2.9)	
Treatment					
Carbo based	41 (41.8)	9 (52.9)	17 (37)	15 (42.9)	0.52
Cis based	57 (58.2)	8 (47.1)	29 (63)	20 (57.1)	
Adjuvant	14 (14.3)	4 (23.5)	5 (10.9)	5 (14.3)	0.39⅟
Neoadjuvant	11 (11.2)	1 (5.9)	7 (15.2)	3 (8.3)	
Lines of treatment for aUC					0.78
1	39 (39.8)	6 (35.3)	20 (43.5)	13 (37.1)	
≥2	59 (60.2)	11 (64.7)	26 (56.5)	22 (62.9)	
Immunotherapy					0.6
Yes	32 (32.7)	7 (41.2)	13 (28.3)	12 (34.3)	
No	66 (67.3)	10 (58.8)	33 (71.7)	23 (65.7)	
Vinflunine					0.19
Yes	19 (19.4)	6 (35.3)	7 (15.2)	6 (17.1)	
No	79 (80.6)	11 (64.7)	39 (84.8)	29 (82.9)	

BMI: body mass index; BSA: body surface area; ⅟ Fisher’s exact test; └ Kruskal–Wallis rank test; ┘ ANOVA; Pearson chi2 test; ECOG: Eastern Cooperative Oncology Group; MSKCC: Memorial Sloan Kettering Cancer Center; *: according to [7]; aUC: advanced urothelial cancer.

**Table 2:** Urothelial cancer-specific survival of the 98 patients included in the analysis: total population and according to genotype.

Characteristic	Total	*p*	ERCC1 19007 Genotypes			*p* ^1^
			C/C	C/T	T/T	
			Median CSS (95% CI)			
Total	22.7 (15.8–30)		41.9 (13.7–NR)	23.2 (9.4–31.8)	21.4 (8.8–30)	0.19
Age at chemo start		0.085				0.046
≤68	30.7 (21–46.5)		30.7 (6.4–NR)	36.9 (9.9–61.4)	22.7 (6.5–NR)	
>68	17.5 (9.5–24.2)		18.8 (7.1–NR)	13.1 (7.2–24.2)	21.4 (5.7–30)	
BMI		0.12				0.052
≤26	17.5 (9.1–22.7)		30.7 (13.7–NR)	10.7 (7.2–24.2)	14.7 (5.6–22.7)	
>26	30 (18.6–38.2)		NR	31 (18.6–54.3)	26.3 (9.5–35.6)	
BSA		0.29				0.33
≤1.9	21 (9.5–28.7)		30.7 (6.4–48.4)	9.9 (6.7–28.7)	22.7 (8.3–35.6)	
>1.9	28.1 (13.7–36.9)		NR	31 (13.1–54.3)	15.8 (5.8–32.7)	
Gender		0.32				0.27
Female	30 (6.5–NR)		11.8 (11.8–NR)	6.7 (5.5–NR)	30 (4.9–NR)	
Male	21.4 (14.7–28.7)		41.9 (13.7–NR)	23.2 (9.4–31)	21 (8.3–26.3)	
Primary site		−0.58				0.64
Bladder	21 (13.1–28.1)		18.8 (7.1–NR)	24.2 (9.4–36.9)	21 (8.3–30)	
Pelvis	30.7 (6.7–41.9)		41.9 (30.7–NR)	7.2 (6.7–NR)	21.4 (21.4–NR)	
Ureter	25.9 (5.8–NR)		-	NR	5.8 (5.8–NR)	
Urethra	NR		NR	NR	-	
ECOG PS		<0.001				0.002
0	28.1 (21.4–38.2)		41.9 (6.4–NR)	38.2 (16.7–54.3)	22.7 (8.8–32.3)	
1	18.6 (9.4–24.2)		21 (7.1–NR)	10.7 (7–28.7)	9.5 (1.2–NR)	
2	5.6 (4.2–NR)		-	5.6 (4.2–NR)	30 (4.9–NR)	
3	1.6 (1.6–NR)		-	-	1.6 (1.6–NR)	
Disease status		0.23				0.41
Distant	21 (9.4–25.9)		18.8 (7.1–NR)	23.2 (7–59)	21 (6.3–25.9)	
Locally advanced/other	28.7 (13.7–36.9)		48.4 (6.4–NR)	18.6 (8.8–31)	32.4 (9.5–NR)	
MSKCC risk *		0.017				0.076
Low	28.7 (16.7–36.9)		48.4 (6.4–NR)	27.2 (9.1–31.8)	32.4 (9.5–NR)	
Intermediate	21 (8.8–25.9)		18.8 (7.1–NR)	23.2 (7–41.1)	21 (6.3–26.3)	
High	5.6 (1.6–NR)		-	NR	5.7 (1.6–NR)	
Treatment						
Carbo based	21 (11.8–31.8)	0.21	17.5 (7.1–NR)	8.8 (5.6–36.9)	23.7 (9.5–32.4)	0.19
Cis based	25.9 (12.6–35.6)		48.4 (6.4–NR)	27.2 (10.7–38.2)	12.6 (5.6–35.6)	
Adjuvant	32.7 (8.3–NR)	0.65	48.4 (30.7–NR)	27.2 (8.3–NR)	32.7 (5.6–NR)	0.86
Neoadjuvant	35.6 (18.6–61.4)		NR	31 (18.6–38.2)	35.6 (12.6–NR)	
Lines of therapy for advanced disease		0.11				0.085
1	9.5 (6.9–28.1)		NR	9.9 (5.6–28.7)	8.3 (2.3–25.9)	
≥2 lines	27.2 (18.8–35.6)		30.7 (13.7–NR)	27.2 (10.7–41.1)	23.7 (15.8–32.7)	
Immunotherapy		0.12				0.08
Yes	30 (17.5–58.9)		30.7 (6.4–NR)	38.2 (8.8–NR)	26.3 (6.5–58.9)	
No	18.8 (9.5–28.1)		48.4 (7.1–NR)	13.1 (7.3–28.7)	14.7 (6.3–32.4)	
Vinflunine		0.3				0.37
Yes	31.8 (21–41.1)		21 (11.8–NR)	31.8 (18.6–NR)	32.4 (12.6–NR)	
No	18.6 (9.5–26.3)		NR	13.1 (8.3–31)	15.8 (6.5–26.3)	

^1^ Stratified for genotypes; BMI: body mass index; BSA: body surface area; ECOG: Eastern Cooperative Oncology Group; MSKCC: Memorial Sloan Kettering Cancer Center; *: according to [7]; NR: not reached.

**Table 3:** Outcomes of the 98 studied patients with advanced urothelial cancer, according to the ERCC1 genotype. Subgroup analysis by immunotherapy received. Survival is measured in months.

No Immunotherapy (*n* = 66)					
	Total	ERCC1 19007 Genotypes			
		C/C	C/T	T/T	*p*
Median CSS	18.8 (9.5–28.1)	48.4 (7.1–NR)	13.1 (7.3–28.7)	14.7 (6.3–32.4)	0.035
Median PFS	7.2 (5.7–10)	7.1 (3.2–NR)	7.5 (5.5–14.2)	6.9 (2.6–13.8)	0.12
Median OS	14.7 (9.4–23.2)	48.4 (7.1–NR)	11.7 (7.3–27.2)	12.6 (5.9–22.7)	0.028
**Immunotherapy (*n* = 32)**					
	**Total**	**ERCC1 19007 genotypes**			
		**C/C**	**C/T**	**T/T**	***p***
Median CSS	30 (17.5–58.9)	30.7 (6.4–NR)	38.2 (8.8–NR)	26.3 (6.5–NR)	0.67
Median PFS	8.3 (5.3–15.5)	5.3 (2.5–15.5)	13.3 (3.8–23.4)	6.3 (3.9–35.8)	0.32
Median OS	24.2 (15.8–41.9)	17.5 (6.4–41.9)	38.2 (16.6–NR)	23.7 (6.5–58.9)	0.45

CSS: cancer-specific survival; PFS: progression-free survival; OS: overall survival; NR: not reached.

## Data Availability

The data presented in this study are available upon reasonable request from the corresponding author. The data are not publicly available.

## Supplementary Materials

The following are available online at https://www.mdpi.com/article/10.3390/curroncol28060380/s1, Figure S1: Patient disposition; Table S1: Detailed description of chemotherapy received as fist-line therapy; Table S2: Progression-free survival for the 98 patients included in the analysis; Table S3: Overall survival for the 98 patients included in the analysis.

## References

[1] Bladder Cancer Statistics. www.wcrf.org.

[2] Sternberg C.N., Yagoda A., Scher H.I., Watson R.C., Herr H.W., Morse M.J., Sogani P.C., Vaughan E.D., Bander N., Weiselberg L.R. (1988). M-VAC (methotrexate, vinblastine, doxorubicin and cisplatin) for advanced transitional cell carcinoma of the urothelium. J. Urol..

[3] Von der Maase H., Hansen S.W., Roberts J.T., Dogliotti L., Oliver T., Moore M.J., Bodrogi I., Albers P., Knuth A., Lippert C.M. (2000). Gemcitabine and cisplatin versus methotrexate, vinblastine, doxorubicin, and cisplatin in advanced or metastatic bladder cancer: Results of a large, randomized, multinational, multicenter, phase III study. J. Clin. Oncol..

[4] Bamias A., Tzannis K., Harshman L.C., Crabb S.J., Wong Y.N., Kumar Pal S., De Giorgi U., Ladoire S., Agarwal N., Yu E.Y. (2019). RISC Investigators. Impact of contemporary patterns of chemotherapy utilization on survival in patients with advanced cancer of the urinary tract: A Retrospective International Study of Invasive/Advanced Cancer of the Urothelium (RISC). Ann. Oncol..

[5] Sternberg C.N., De Mulder P.H., Schornagel J.H., Theodore C., Fossa S.D., Van Oosterom A.T., Witjes F., Spina M., Van Groeningen C.J., De Balincourt C. (2001). European Organization for Research and Treatment of Cancer Genitourinary Tract Cancer Cooperative Group. Randomized phase III trial of high-dose-intensity methotrexate, vinblastine, doxorubicin, and cisplatin (MVAC) chemotherapy and recombinant human granulocyte colony-stimulating factor versus classic MVAC in advanced urothelial tract tumors: European Organization for Research and Treatment of Cancer Protocol no. 30924. J. Clin. Oncol..

[6] Bamias A., Dafni U., Karadimou A., Timotheadou E., Aravantinos G., Psyrri A., Xanthakis I., Tsiatas M., Koutoulidis V., Constantinidis C. (2013). Prospective, open-label, randomized, phase III study of two dose-dense regimens MVAC versus gemcitabine/cisplatin in patients with inoperable, metastatic or relapsed urothelial cancer: A Hellenic Cooperative Oncology Group study (HE 16/03). Ann. Oncol..

[7] Bajorin D.F., Dodd P.M., Mazumdar M., Fazzari M., McCaffrey J.A., Scher H.I., Herr H., Higgins G., Boyle M.G. (1999). Long-term survival in metastatic transitional-cell carcinoma and prognostic factors predicting outcome of therapy. J. Clin. Oncol..

[8] Bamias A., Tzannis K., Bamia C., Harshman L.C., Crabb S., Plimack E.R., Pal S., De Giorgi U., Ladoire S., Theodore C. (2019). The Impact of Cisplatin- or Non-Cisplatin-Containing Chemotherapy on Long-Term and Conditional Survival of Patients with Advanced Urinary Tract Cancer. Oncologist.

[9] Bellmunt J., De Wit R., Vaughn D.J., Fradet Y., Lee J.L., Fong L., Vogelzang N.J., Climent M.A., Petrylak D.P., Choueiri T.K. (2017). KEYNOTE-045 Investigators. Pembrolizumab as Second-Line Therapy for Advanced Urothelial Carcinoma. N. Engl. J. Med..

[10] Balar A.V., Galsky M.D., Rosenberg J.E., Powles T., Petrylak D.P., Bellmunt J., Loriot Y., Necchi A., Hoffman-Censits J., Perez-Gracia J.L. (2017). IMvigor Study Group. Atezolizumab as first-line treatment in cisplatin-ineligible patients with locally advanced and metastatic urothelial carcinoma: A single-arm, multicentre, phase 2 trial. Lancet.

[11] Sharma P., Retz M., Siefker-Radtke A., Baron A., Necchi A., Bedke J., Plimack E.R., Vaena D., Grimm M.O., Bracarda S. (2017). Nivolumab in metastatic urothelial carcinoma after platinum therapy (CheckMate 275): A multicentre, single-arm, phase 2 trial. Lancet Oncol..

[12] Powles T., Rosenberg J.E., Sonpavde G.P., Loriot Y., Duran I., Lee J.L., Matsubara N., Vulsteke C., Castellano D., Wu C. (2021). EnfortumabVedotin in Previously Treated Advanced Urothelial Carcinoma. N. Engl. J. Med..

[13] Loriot Y., Necchi A., Park S.H., Garcia-Donas J., Huddart R., Burgess E., Fleming M., Rezazadeh A., Mellado B., Varlamov S. (2019). 348 BLC2001 Study Group. Erdafitinib in Locally Advanced or Metastatic Urothelial Carcinoma. N. Engl. J. Med..

[14] Reardon J.T., Sancar A. (2005). Nucleotide excision repair. Prog. Nucleic Acid. Res. Mol. Biol..

[15] Hanawalt P.C. (2002). Subpathways of nucleotide excision repair and their regulation. Oncogene.

[16] Rabik C.A., Dolan M.E. (2007). Molecular mechanisms of resistance and toxicity associated with platinating agents. Cancer Treat. Rev..

[17] Altaha R., Liang X., Yu J.J., Reed E. (2004). Excision repair cross complementing-group 1: Gene expression and platinum resistance. Int. J. Mol. Med..

[18] Arriagada R., Bergman B., Dunant A., Le Chevalier T., Pignon J.P., Vansteenkiste J. (2004). Cisplatin-based adjuvant chemotherapy in patients with completely resected non-small-cell lung cancer. N. Engl. J. Med..

[19] Olaussen K.A., Dunant A., Fouret P., Brambilla E., Andre F., Haddad V., Taranchon E., Filipits M., Pirker R., Popper H.H. (2006). DNA repair by ERCC1 in non-small-cell lung cancer and cisplatin-based adjuvant chemotherapy. N. Engl. J. Med..

[20] Kim K.H., Do I.G., Kim H.S., Chang M.H., Kim H.S., Jun H.J., Uhm J., Yi S.Y., Lim D.H., Ji S.H. (2010). Excision repair cross-complementation group 1 (ERCC1) expression in advanced urothelial carcinoma patients receiving cisplatin-based chemotherapy. APMIS.

[21] Hoffmann A.C., Wild P., Leicht C., Bertz S., Danenberg K.D., Danenberg P.V., Stohr R., Stockle M., Lehmann J., Schuler M. (2010). MDR1 and ERCC1 expression predict outcome of patients with locally advanced bladder cancer receiving adjuvant chemotherapy. Neoplasia.

[22] Bellmunt J., Paz-Ares L., Cuello M., Cecere F.L., Albiol S., Guillem V., Gallardo E., Carles J., Mendez P., De la Cruz J.J. (2007). Gene expression of ERCC1 as a novel prognostic marker in advanced bladder cancer patients receiving cisplatin-based chemotherapy. Ann. Oncol..

[23] Nikitas N., Karadimou A., Tsitoura E., Soupos N., Tsiatas M., Karavasilis V., Pectasides D., Pavlidis N., Chrisofos M., Adamakis I. (2012). Association of ERCC1 SNPs with outcome in platinum-treated patients with advanced urothelial cancer: A Hellenic Cooperative Oncology Group study. Pharmacogenomics.

[24] Stoehlmacher J., Park D.J., Zhang W., Yang D., Groshen S., Zahedy S., Lenz H.J. (2004). A multivariate analysis of genomic polymorphisms: Prediction of clinical outcome to 5-FU/oxaliplatin combination chemotherapy in refractory colorectal cancer. Br. J. Cancer.

[25] Matuszczak M., Salagierski M. (2020). Diagnostic and Prognostic Potential of Biomarkers CYFRA 21.1, ERCC1, p53, FGFR3 and TATI in Bladder Cancers. Int. J. Mol. Sci..

[26] Horwich A., Babjuk M., Bellmunt J., Bruins H.M., De Reijke T.M., De Santis M., Gillessen S., James N., Maclennan S., Palou J. (2019). EAU-ESMO consensus statements on the management of advanced and variant bladder cancer-an international collaborative multi-stakeholder effort: Under the auspices of the EAU and ESMO Guidelines Committees. Ann. Oncol..

[27] Bamias A., Merseburger A.S., Loriot Y., James N., Choy E., Castellano D., Lopez-Rios F., Calabro F., Kramer M., De Velasco G. (2021). SAUL, a single-arm study of atezolizumab for chemotherapy-pretreated locally advanced or metastatic carcinoma of the urinary tract: Outcomes by key baseline factors, PD-L1 expression and prior platinum therapy. ESMO Open.

[28] Aiello M.M., Solinas C., Santoni M., Battelli N., Restuccia N., Latteri F., Paratore S., Verderame F., Albanese G.V., Bruzzi P. (2020). Excision Repair Cross Complementation Group 1 Single Nucleotide Polymorphisms and Nivolumab in Advanced Non-Small Cell Lung Cancer. Front. Oncol..

[29] Nikanjam M., Arguello D., Gatalica Z., Swensen J., Barkauskas D.A., Kurzrock R. (2020). Relationship between protein biomarkers of chemotherapy response and microsatellite status, tumor mutational burden and PD-L1 expression in cancer patients. Int. J. Cancer.

[30] Xu Z.C., Cai H.Z., Li X., Xu W.Z., Xu T., Yu B., Zou Q., Xu L. (2016). ERCC1 C118T polymorphism has predictive value for platinum-based chemotherapy in patients with late-stage bladder cancer. Genet. Mol. Res..

[31] Galsky M.D., Saci A., Szabo P.M., Han G.C., Grossfeld G., Collette S., Siefker-Radtke A., Necchi A., Sharma P. (2020). Nivolumab in Patients with Advanced Platinum-resistant Urothelial Carcinoma: Efficacy, Safety, and Biomarker Analyses with Extended Follow-up from CheckMate 275. Clin. Cancer Res..

[32] Powles T., Park S.H., Voog E., Caserta C., Valderrama B.P., Gurney H., Kalofonos H., Radulovic S., Demey W., Ullen A. (2020). Avelumab Maintenance Therapy for Advanced or Metastatic Urothelial Carcinoma. N. Engl. J. Med..

