# Supplementary material for: ERCC1 19007 Polymorphism in Greek Patients with Advanced Urothelial Cancer Treated with Platinum-Based Chemotherapy: Effect of the Changing Treatment Paradigm: A Cohort Study by the Hellenic GU Cancer Group
Source: Curr Oncol. 2021 Nov 5;28(6):380. doi: 10.3390/curroncol28060380 (PMC8653964; doi:10.3390/curroncol28060380)
Supplement: Supplementary file 1 [file curroncol-28-00380-s001.zip › curroncol-1400790-supplementary.pdf]

Current Oncology

Article

ERCC1 19007 Polymorphism in Greek Patients with Advanced Urothelial Cancer Treated with Platinum-Based Chemotherapy: Effect of the Changing Treatment Paradigm. A Cohort Study by the Hellenic GU Cancer Group

Aristotelis Bamias [1,2,*], Konstantinos Koutsoukos [2,3], Nikos Gavalas [2,3], Roubini Zakopoulou [1,2], Kimon Tzannis [1,2], Nikos Dedes [3], Anna Boulouta [1], Charalampos Fragkoulis [2,4], Eythymios Kostouros [5], Athanasios Dellis [6], Iraklis Mitsogiannis [7], Ioannis Adamakis [8], Ioannis Anastasiou [8], Andreas Skolarikos [7], Athanasios Papatsoris [7], Konstantinos Stravodimos [8], Nikolaos Ferrakis [9], Stamatina Pagoni [5], Konstantinos Ntoumas [2,4], Dionysios Mitropoulos [8], Charalambos Deliveliotis [7], Constantinos A. Constantinides [8] and Meletios A. Dimopoulos [2,3]

Supplementary Material

Figure S1. Patient disposition.

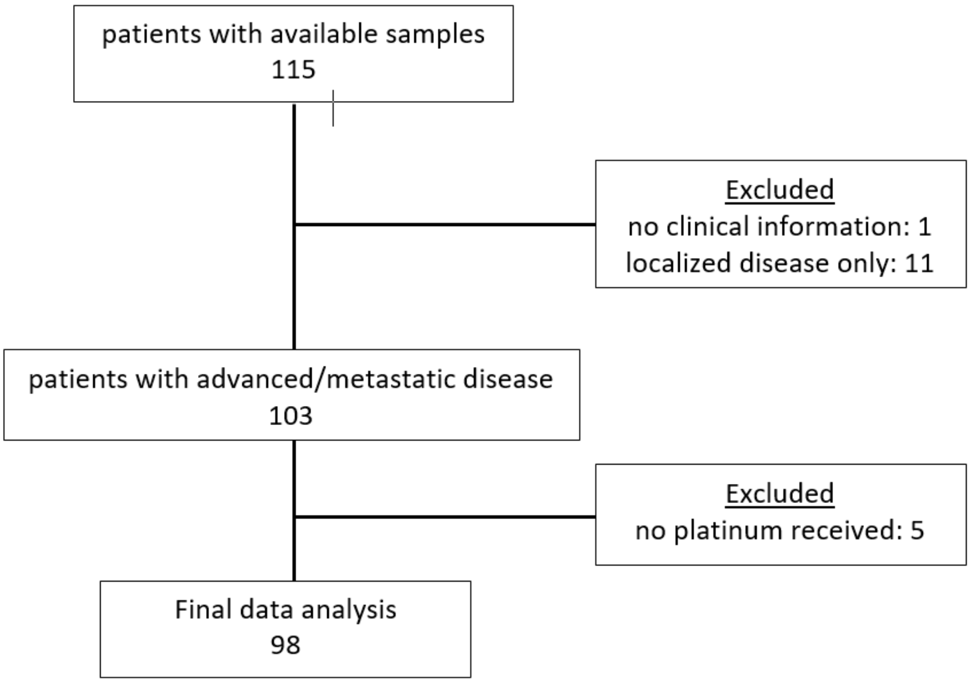

Table S1. Detailed description of chemotherapy received as 1st-line therapy.

Group	Regimen	n	%
Cisplatin-based	DD GC[1]	7	
	HD MVAC[1]	1	
	MVAC[2]	4	
	GC[3]	34	
	GC-Atezolizumab[3]	2	
Carboplatin-based	MCaVi[4]	4	
	GCa[3]	34	
	GCa-Atezolizumab[3]	2	
Other	Vinflunine[5]	3	
	Docetaxel-Ramucirumab/Placebo[6]	3	
	Durvalumab[7]	2	
	Atezolizumab[3]	2	

DD: dose dense; GC: gemcitabine/cisplatin; MVAC: methotrexate, vinblastine, doxorubicin, cisplatin; MCaVi: methotrexate, carboplatin, vinblastine; GCa: gemcitabine/carboplatin.

Table S2. Progression free Survival for 98 patients included in the analysis.

	**Total**		**ERCC1 19007 genotypes**			
		p	**C/C**	**C/T**	**T/T**	***p****
				Median PFS (95% CI)		
Total	7.3 (6.2-10)		7.1 (3.6-33)	8 (6.2-15)	6.9 (4.6-12.2)	.59
Age at chemo start						
≤68	10 (7.1-19.8)	.053	7.1 (3.6-NR)	16.5 (7.5-28.1)	6.9 (2.9-31.5)	.028
>68	6.3 (4.9-8.5)		5.7 (2.6-NR)	6.2 (4.6-10)	6.3 (2.7-13.8)	
BMI						
≤26	6.1 (3.8-9.5)	.056	9.5 (2.5-33)	6.2 (3.8-9.9)	5.6 (2.3-12.2)	.065
>26	8.5 (7.1-18.7)		5.7 (2.6-NR)	14.2 (7.3-20.8)	7.2 (5.6-26.7)	
BSA						
≤1.9	6.3 (5.3-10)	.41	5.3 (2.5-27.4)	7.5 (4.5-14.2)	6.3 (3.7-14.7)	.52
>1.9	7.3 (6.8-16.8)		7.1 (3.6-NR)	10 (6.9-18.7)	7.1 (2.3-20.4)	
Gender						
Female	5.5 (2.6-52.9)	.36	2.6 (2.6-NR)	5.5 (2.8-NR)	3.9 (0.5-NR)	.23
Male	7.3 (6.3-12.2)		7.1 (3.6-27.4)	8 (6.8-15.1)	6.9 (5.6-12.2)	
Primary site						
Bladder	7.3 (6.2-13.8)		5.3 (3.2-NR)	8 (6.8-16.8)	6.9 (4.6-14.7)	
Pelvis	8 (2.5-15.5)	.13	15.5 (2.5-NR)	3.8 (2.8-NR)	12.2 (6.1-NR)	.15
Ureter	2.3 (1.8-NR)		-	NR	1.8 (1.8-NR)	
Urethra	7.1 (2.1-NR)		7.1 (7.1-NR)	NR	-	
ECOG PS						
0	8.5 (6.8-15.5)		15.5 (2.5-34.5)	8.3 (5.8-20.8)	7.2 (5.6-13.8)	
1	7.1 (5.6-14.2)	<.001	4.9 (2.6-NR)	8 (5.5-16.8)	6.9 (1.2-NR)	.006
2	2.8 (0.5-NR)		-	2.8 (1.7-NR)	3.9 (0.5-NR)	
3	1.6 (1.6-NR)		-	-	1.6 (1.6-NR)	
Disease status						
Distant	6.8 (4.8-8)	.035	4.9 (2.6-9.5)	8 (6.8-18.7)	5.6 (2.7-7.1)	.074
Locally advanced/other	14.2 (6.2-19.8)		27.4 (2.5-NR)	7.3 (4.9-16.5)	20.4 (5.6-35.8)	
MSKCC risk*						
Low	14.7 (7.2-20.4)	<.001	27.4 (2.5-NR)	9.9 (5.5-16.5)	20.4 (5.6-35.8)	.002
Intermediate	6.9 (5.6-8)		4.9 (2.6-9.5)	8 (4.8-18.7)	6.3 (2.7-10)	
High	2.6 (1.6-NR)		-	NR	2.6 (1.6-NR)	
Treatment						
Carbo based	6.1 (3.9-9.5)	.071	4.9 (2.5-34.5)	4.8 (2.8-10)	8.5 (3.9-14.7)	.044
Cis based	8 (6.9-16.5)		15.5 (3.6-NR)	13.3 (7.3-19.8)	5.6 (2.3-16.8)	

Adjuvant	24.4 (6.9-34.5)	.6	15.5 (4.9-NR)	24.4 (8-NR)	26.7 (5.6-NR)	.64
Neoadjuvant	19.8 (13.3-33)		NR	19.8 (13.3-28.7)	13.8 (7.2-NR)	
Lines of therapy for advanced disease		.32				.45
1	7 (5.3-14.7)		5.7 (4.9-NR)	7.3 (4.6-19.8)	5.6 (1.8-14.7)	
≥2	8 (6.3-13.8)		7.1 (2.6-27.4)	8 (4.9-16.5)	7.2 (4.6-16.8)	
Immunotherapy		.43				.3
Yes	8.3 (5.3-15.5)		5.3 (2.5-15.5)	13.3 (3.8-23.3)	6.3 (3.9-35.8)	
No	7.2 (5.7-15.5)		7.1 (3.2-NR)	7.5 (5.5-14.2)	6.9 (2.6-13.8)	
Vinflunine		.91				.98
Yes	10 (6.1-20.4)		3.6 (2.6-NR)	16.5 (2.1-28.7)	10 (6.1-NR)	
No	7 (5.6-9.5)		9.5 (3.2-NR)	7.3 (5.5-14.2)	6.3 (2.9-12.2)	

*Stratified for genotypes; BMI: Body mass index; BSA: body surface area; ECOG: Eastern Co-operative Oncology Group; MSKCC: Memorial Sloan Kettering Cancer Center; *: according to ref 7; NR: not reached.

Table S3. Overall Survival for 98 patients included in the analysis.

	**Total**		**ERCC1 19007 genotypes**			
		p	**C/C**	**C/T**	**T/T**	*p**
			Median OS (95% CI)			
Total	19.8 (12.6-26.3)		30.7 (11.8-NR)	18.6 (9.1-28.7)	19.8 (8.3-25.9)	.31
Age at chemo start						
≤68	25.9 (11.7-32.7)	.3	30.7 (6.4-NR)	28.1 (9.9-41.1)	19.8 (6.5-32.7)	.23
>68	16.7 (9.1-23.7)		18.8 (7.1-NR)	13.1 (7.2-24.2)	15.8 (5.7-30)	
Bmi						
≤26	16.7 (8.8-21)	.21	30.7 (13.7-NR)	10.7 (7.2-24.2)	8.3 (5.6-21.4)	.108
>26	27.2 (12.6-32.7)		11.8 (6.4-NR)	27.2 (9.4-38.2)	26.3 (9.5-35.6)	
BSA						
≤1.9	17.5 (9.1-24.2)	.36	18.8 (6.4-48.4)	9.9 (6.7-24.2)	21 (6.5-30)	.39
>1.9	23.2 (12.6-32.4)		48.9 (7.1-NR)	28.1 (11.7-36.9)	12.6 (5.8-32.4)	
Gender						
Female	30 (6.5-NR)	.21	11.8 (11.8-NR)	6.7 (5.5-NR)	30 (4.9-NR)	.15
Male	18.8 (11.7-24.2)		30.7 (7.1-48.9)	18.6 (9.4-28.1)	14.7 (7.7-22.7)	
Primary site						
Bladder	17.5 (10.7-24.2)		17.5 (6.8-NR)	18.6 (9.4-28.7)	14.7 (7.7-30)	
Pelvis	21.4 (6.7-32.4)	.79	41.9 (30.7-NR)	7.2 (6.7-NR)	21.4 (19.8-NR)	.77
Ureter	25.9 (5.8-NR)		-	NR	5.8 (5.8-NR)	
Urethra	48.9 (18.6-NR)		48.9 (NR)	NR	-	
ECOG PS						
0	25.9 (17.5-32.4)		41.9 (6.4-NR)	28.1 (11.7-46.5)	21.4 (8.8-26.3)	
1	14.7 (8.3-22.8)	<.001	18.8 (3.7-NR)	10.7 (7-24.2)	9.5 (1.2-NR)	.002
2	5.6 (4.2-NR)		-	5.6 (4.2-NR)	30 (4.9-NR)	
3	1.6 (1.6-NR)		-	-	1.6 (1.6-NR)	
Disease status						
Distant	17.5 (8.3-23.7)	.17	17.5 (6.8-NR)	23.2 (7-59)	8.8 (5.9-23.7)	.3
Locally advanced/other	22.8 (13.1-31.8)		48.4 (6.4-NR)	16.7 (8.8-28.7)	32.4 (9.5-59)	
MSKCC risk*						
Low	27.2 (13.7-32.4)	.028	48.4 (6.4-NR)	18.6 (9.1-28.6)	32.4 (9.5-NR)	.092
Intermediate	18.6 (8.8-23.7)		17.5 (6.8-NR)	23.2 (7-41.1)	15.8 (5.9-25.9)	
High	5.6 (1.6-NR)		-	NR	5.7 (1.6-NR)	
Treatment						

Carbo based	18.8 (8.8-26.3)	.28	17.5 (6.8-NR)	8.8 (5.6-36.9)	21.4 (8.8-30)	.29
Cis based	22.7 (10.7-29.6)		48.4 (6.4-NR)	22.8 (9.9-29.6)	8.3 (5.6-35.6)	
Adjuvant	30.7 (8.3-NR)	.77	30.7 (6.8-NR)	27.2 (8.3-NR)	32.7 (5.6-NR)	.94
Neoadjuvant	35.6 (18.6-59)		NR	31 (18.6-38.2)	35.6 (12.6-NR)	
Lines of therapy for advanced disease		.12				.102
1	8.3 (6.4-23.2)		7.1 (6.4-NR)	8.3 (5.5-28.1)	8.3 (2.3-25.9)	
≥2	23.7 (18.6-31.8)		30.7 (13.7-48.9)	24.2 (10.7-38.2)	21 (8.8-32.4)	
Immunotherapy		.073				.058
Yes	24.2 (15.8-41.9)		17.5 (6.4-41.9)	38.2 (8.8-NR)	23.7 (6.5-58.9)	
No	14.7 (9.4-23.2)		48.4 (7.1-NR)	11.7 (7.3-27.2)	12.6 (5.9-22.7)	
Vinflunine		.43				.5
Yes	29.6 (19.8-35.6)		21 (11.8-NR)	29.6 (18.6-41.1)	21 (12.6-NR)	
No	15.8 (8.8-23.2)		41.9 (6.8-NR)	11.7 (7.3-24.2)	14.7 (6.3-25.9)	

*Stratified for genotypes; BMI: Body mass index; BSA: body surface area; ECOG: Eastern Co-operative Oncology Group; MSKCC: Memorial Sloan Kettering Cancer Center; *: according to ref 7; NR: not reached.

References

1. Bamias, A.; Dafni, U.; Karadimou, A.; Timotheadou, E.; Aravantinos, G.; Psyrri, A.; Xanthakis, I.; Tsiatas, M.; Koutoulidis, V.; Constantinidis, C.; et al. Prospective, open-label, randomized, phase III study of two dose-dense regimens MVAC versus gemcitabine/cisplatin in patients with inoperable, metastatic or relapsed urothelial cancer: a Hellenic Cooperative Oncology Group study (HE 16/03). *Ann. Oncol.* **2013**, *24*, 1011–1017
2. Bamias, A.; Aravantinos, G.; Deliveliotis, C.; Bafaloukos, D.; Kalofonos, C.; Xiros, N.; Zervas, A.; Mitropoulos, D.; Samantas, E.; Pectasides, D.; et al. Hellenic Cooperative Oncology Group. Docetaxel and cisplatin with granulocyte colony-stimulating factor (G-CSF) versus MVAC with G-CSF in advanced urothelial carcinoma: a multicenter, randomized, phase III study from the Hellenic Cooperative Oncology Group. *J. Clin. Oncol.* **2004**, *22*, 220–228.
3. Galsky, M.D.; Arija, J.Á.A.; Bamias, A.; Davis, I.D.; De Santis, M.; Kikuchi, E.; Garcia-Del-Muro, X.; De Giorgi, U.; Mencinger, M.; Izumi, K.; et al. Atezolizumab with or without chemotherapy in metastatic urothelial cancer (IMvigor130): a multicentre, randomised, placebo-controlled phase 3 trial. *Lancet* **2020**, *395*, 1547–1557.
4. De Santis, M.; Bellmunt, J.; Mead, G.; Kerst, J.M.; Leahy, M.; Maroto, P.; Gil, T.; Marreaud, S.; Daugaard, G.; Skoneczna, I.; et al. Randomized phase II/III trial assessing gemcitabine/carboplatin and methotrexate/carboplatin/vinblastine in patients with advanced urothelial cancer who are unfit for cisplatin-based chemotherapy: EORTC study 30986. *J. Clin. Oncol.* **2012**, *30*, 191–199.
5. Petrylak, D.P.; de Wit, R.; Chi, K.N.; Drakaki, A.; Sternberg, C.N.; Nishiyama, H.; Castellano, D.; Hussain, S.; Fléchon, A.; Bamias, A.; et al. Ramucirumab plus docetaxel versus placebo plus docetaxel in patients with locally advanced or metastatic urothelial carcinoma after platinum-based therapy (RANGE): a randomised, double-blind, phase 3 trial. *Lancet* **2017**, *390*, 2266–2277
6. Pistamaltzian, N.; Tzannis, K.; Pissanidou, V.; Peroukidis, S.; Milaki, G.; Karavasilis, V.; Mitsogiannis, I.; Varkarakis, I.; Papatsoris, A.; Dellis, A.; et al. Treatment of relapsed urothelial bladder cancer with vinflunine: real-world evidence by the Hellenic Genitourinary Cancer Group. *Anticancer Drugs.* **2016**, *27*, 48–53.
7. Powles, T.; van der Heijden, M.S.; Castellano, D.; Galsky, M.D.; Loriot, Y.; Petrylak, D.P.; Ogawa, O.; Park, S.H.; Lee, J.L.; De Giorgi, U.; et al. Durvalumab alone and durvalumab plus tremelimumab versus chemotherapy in previously untreated patients with unresectable, locally advanced or metastatic urothelial carcinoma (DANUBE): a randomised, open-label, multicentre, phase 3 trial. *Lancet Oncol.* **2020**, *21*, 1574–1588.
